# A prospective observational study on impact of epinephrine administration route on acute myocardial infarction patients with cardiac arrest in the catheterization laboratory (iCPR study)

**DOI:** 10.1186/s13054-022-04275-8

**Published:** 2022-12-20

**Authors:** Ali Aldujeli, Ayman Haq, Kristen M. Tecson, Zemyna Kurnickaite, Karolis Lickunas, Som Bailey, Vacis Tatarunas, Rima Braukyliene, Giedre Baksyte, Montazar Aldujeili, Hussein Khalifeh, Kasparas Briedis, Rasa Ordiene, Ramunas Unikas, Anas Hamadeh, Emmanouil S. Brilakis

**Affiliations:** 1 Hospital of Lithuanian University of Health Sciences Kaunas Clinics, Kaunas, Lithuania; 2 Institute of Cardiology, Lithuanian University of Health Sciences, Kaunas, Lithuania; 3 Abbott Northwestern Hospital/Minneapolis Heart Institute Foundation, Minneapolis, MN USA; 4 Baylor Scott & White Research Institute, Dallas, TX USA; 5 Medical City Fort Worth, Fort Worth, TX USA; 6 Republican Hospital of Panevezys, Panevezys, Lithuania; 7 University of Brescia, Brescia, Italy; 8 Kreiskrankenhaus Rotenburg, Rotenburg an der Fulda, Germany; 9 Texas Cardiovascular Institute, Fort Worth, TX USA

**Keywords:** Intracoronary epinephrine, Cardiopulmonary resuscitation, Cardiac arrest, Acute myocardial infarction, Stent thrombosis, Return of spontaneous circulation (ROSC)

## Abstract

**Background:**

Epinephrine is routinely utilized in cardiac arrest; however, it is unclear if the route of administration affects outcomes in acute myocardial infarction patients with cardiac arrest.

**Objectives:**

To compare the efficacy of epinephrine administered via the peripheral intravenous (IV), central IV, and intracoronary (IC) routes.

**Methods:**

Prospective two-center pilot cohort study of acute myocardial infarction patients who suffered cardiac arrest in the cardiac catheterization laboratory during percutaneous coronary intervention. We compared the outcomes of patients who received epinephrine via peripheral IV, central IV, or IC.

**Results:**

158 participants were enrolled, 48 (30.4%), 50 (31.6%), and 60 (38.0%) in the central IV, IC, and peripheral IV arms, respectively. Peripheral IV epinephrine administration route was associated with lower odds of achieving return of spontaneous circulation (ROSC, odds ratio = 0.14, 95% confidence interval = 0.05–0.36, *p* < 0.0001) compared with central IV and IC administration. (There was no difference between central IV and IC routes; *p* = 0.9343.) The odds of stent thrombosis were significantly higher with the IC route (IC vs. peripheral IV OR = 4.6, 95% CI = 1.5–14.3, *p* = 0.0094; IC vs. central IV OR = 6.0, 95% CI = 1.9–19.2, *p* = 0.0025). Post-ROSC neurologic outcomes were better for central IV and IC routes when compared with peripheral IV.

**Conclusion:**

Epinephrine administration via central IV and IC routes was associated with a higher rate of ROSC and better neurologic outcomes compared with peripheral IV administration. IC administration was associated with a higher risk of stent thrombosis.

*Trial registration *This trial is registered at NCT05253937.

**Graphical Abstract:**

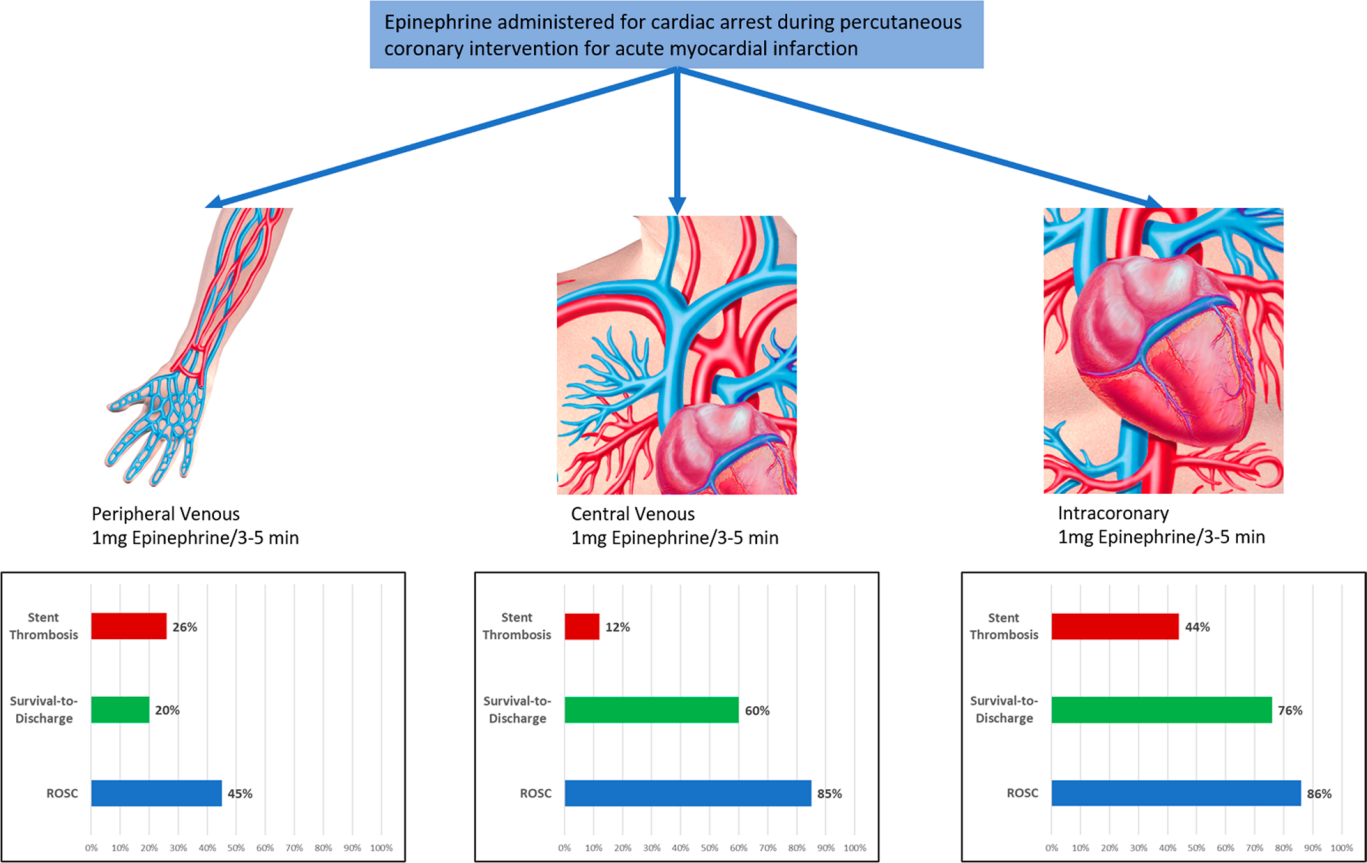

## Introduction

In-hospital cardiac arrest (IHCA) is a major challenge experienced by all healthcare systems worldwide [1, 2]. Despite significant progress in cardiopulmonary resuscitation (CPR) in recent years, outcomes remain poor, with only approximately 49% survival to hospital discharge [3]. Epinephrine administration remains a cornerstone in the treatment of IHCA [4]. However, the optimal administration route remains controversial [5, 6]. Various routes of administration, including intravenous, intramuscular, intraosseous, and endotracheal routes, have been studied [7–9]. Initially, the American guidelines for the treatment of IHCA recommended injection of 0.5 mg of epinephrine directly into the right ventricle via a parasternal approach, aiming to rapidly achieve higher peak intracardiac concentrations and a more central effect; however, the intravenous route remains preferred due to feasibility and safety [5, 6, 10].

The incidence of cardiac arrest in the cardiac catheterization laboratory is relatively low, approximately 1% during the past few decades [11]. However, with approximately 1 million percutaneous coronary procedures performed every year in the USA, roughly 10,000 patients will suffer in-catheterization laboratory cardiac arrest annually [12]. Arrest in the cardiac catheterization laboratory allows intracoronary (IC) epinephrine administration as part of resuscitative efforts. To our knowledge, IC epinephrine administration for intraprocedural cardiac arrest has not been compared with other routes of administration. The purpose of the present study was to compare the efficacy of peripheral IV, central IV, and IC epinephrine administration in achieving the return of spontaneous circulation (ROSC) in a cohort of acute myocardial infarction patients who underwent primary percutaneous coronary intervention and experienced cardiac arrest in the cardiac catheterization laboratory.

## Methods

### Study design

This was a prospective two-center pilot cohort study conducted in the Hospital of the Lithuanian University of Health Sciences Kaunas Clinics and the Republican Hospital of Panevezys. Both cardiac centers cover six out of ten administrative regions in the republic of Lithuania. The study enrolled acute myocardial infarction patients who suffered a cardiac arrest in the cardiac catheterization laboratory during percutaneous coronary intervention. Cardiac resuscitation was performed according to the European Resuscitation Council (ERC) Guidelines [13]. Because the preferred route of epinephrine administration is through a central venous catheter (via internal jugular or subclavian vein), it was the first choice for epinephrine administration when available [6]. In cases where a central venous catheter had not been placed, the route of epinephrine administration (peripheral IV catheter or IC catheter) during cardiac arrest was left to the discretion of the treating physician.

### Study inclusion and exclusion criteria

The study included patients from April 1, 2018, to June 1, 2021, aged 18 years or older with either non-ST elevation myocardial infarction (NSTEMI) or ST elevation myocardial infarction (STEMI) who received dual antiplatelet therapy (acetylsalicylic acid 300 mg and ticagrelor 180 mg), or triple therapy (oral anticoagulant, acetylsalicylic acid 300 mg and clopidogrel 300 or 600 mg) at least 30 min prior to primary percutaneous intervention and had a cardiac arrest during their procedure.

Patients were excluded from the study if cardiac arrest occurred prior to being transported to the cardiac catheterization laboratory. Similarly, patients who suffered cardiac arrest for less than 60 s were excluded as they would not have received epinephrine in this timeframe. Those who presented with a rhythm other than sinus rhythm or atrial fibrillation/flutter or received vasopressor and/or antiarrhythmic medication prior to CPR were excluded to limit confounding by medications given prior to CPR. Patients who required mechanical circulatory support, had a concomitant acute illness (infection, etc.), significant comorbid disease (liver disease, end-stage renal failure, solid organ malignancy), prior coronary artery disease, or underwent primary fibrinolysis were excluded to limit the study to those who would have a similar prognosis in the event of cardiac arrest [14]. Patients who received targeted temperature management post-CPR were excluded as it is not the standard of care in in-hospital cardiac arrest [15]. Lastly, those with an allergy to contrast media were also excluded.

### Data collection

Data collected included patient demographics such as age, sex, body mass index, primary diagnosis, clinical history, and comorbidities. Cardiac rhythm on admission and prior to cardiac arrest were recorded. In addition to routine laboratory tests, additional blood samples were drawn into vacutainer tubes (Greiner Bio-One Vacuette North America, Inc., Monroe, NC) that contained 3.2% sodium citrate for measurement of platelet aggregation with adenosine diphosphate (ADP) and international normalized ratio (INR) values. Data such as time-to-first epinephrine administration, the route of administration of epinephrine, the total dose of epinephrine administered, the total number of electric cardioversions attempted during the cardiac arrest, and the total time of resuscitation until ROSC or death were recorded. Furthermore, post-resuscitation left ventricular ejection fraction (LVEF), clinical course (stent thrombosis, KILLIP classification, length of stay in CCU, and ICD implantation), short-term outcomes (in-hospital death), and neurological outcomes (cerebral performance category (CPC)) were recorded [16].

### Study endpoints

The primary endpoint was (the rate of) ROSC. In-hospital stent thrombosis was the secondary endpoint, and survival-to-discharge with favorable neurologic status (CPC score 1–2) was the tertiary endpoint [16].

#### Definition

Cardiac arrest was defined as a sudden cessation of cardiac function, precipitated by ventricular fibrillation (VF), pulseless electrical activity (PEA), or asystole requiring CPR [5, 6]. Time-to-epinephrine administration and time-to-ROSC were measured in minutes starting from the initiation of CPR until the first epinephrine dose and first ROSC, respectively. ROSC was defined as the return of spontaneous sustained cardiac activity for more than three consecutive minutes. STEMI and NSTEMI were defined according to the fourth universal definition of myocardial infarction [17]. Door-to-cath laboratory time was defined as the time (in minutes) from first medical contact at the facility to reaching the catheterization laboratory. Dyslipidemia was defined as a fasting total cholesterol level of more than 100 mg/dl or the use of lipid-lowering medications [18]. Hypertension was defined as systolic blood pressure higher than or equal to 130 mmHg and diastolic higher than 80 mmHg or the use of blood pressure-lowering medication [19]. Electrolyte imbalance was defined as abnormal potassium (< 3.6 mEq/L or > 5.2 mEq/L) or abnormal magnesium (< 1.3 mEq/L or > 2.1 mEq/L) just prior to catheterization. Obesity was defined as having a body mass index (BMI) over 30 kg/m^2^. Diabetes mellitus was defined as a fasting plasma glucose level ≥ 126 mg/dL, or the use of blood glucose-lowering medication [20]. Cardiogenic shock was defined as in-hospital use of vasopressors or persistent hypotension with evidence of hypo-perfusion caused by severe cardiac dysfunction despite adequate fluid administration [21]. Successful PCI was defined as the implantation of a second-generation drug-eluting stent resulting in the reduction in a coronary artery lesion to less than 20%. In-stent thrombosis was defined as new ST elevation with anginal symptoms or an equivalent due to thrombotic occlusion of the stent placed at the culprit lesion confirmed by coronary angiography during the index hospitalization. All patients who developed in-stent thrombosis were additionally treated with a GP IIb/IIIa inhibitor according to ESC guidelines [22]. Linear measurements of cardiac chambers and LVEF were obtained according to the recommendations of the European Association of Cardiovascular Imaging [23].

### Statistical analysis

Categorical variables are presented as frequencies and percentages. Most continuous variables were skewed and are presented as median [quartile 1, quartile 3]. Differences in patient characteristics between epinephrine administration routes were assessed by chi-square (or Fisher’s exact test) and Kruskal–Wallis tests (or analysis of variance), as appropriate. We created multivariable logistic regression models via stepwise selection (which was confirmed via backward and forward selection) to preserve degrees of freedom, to investigate the association of epinephrine route with the outcomes of interest (i.e., ROSC, in-stent thrombosis, hospital survival with favorable neurologic status) while accounting for potential confounders (i.e., heart rhythm prior to CPR, age, and baseline serum potassium, hemoglobin, and LVEF) identified as having significant associations with epinephrine administration route (Table 1). We additionally tested for associations in time-to-ROSC across treatment groups and between neurologic outcomes (favorable or not) via the Kruskal–Wallis test.

**Table 1:** Characteristics of acute myocardial infarction patients undergoing cardiopulmonary resuscitation by epinephrine administration route

		Epinephrine administration route			
Characteristic	Overall (*n* = 158)	Central IV (*n* = 48)	Intracoronary (*n* = 50)	Peripheral (*n* = 60)	*p* value
Sex (male)	88 (56%)	28 (58%)	29 (58%)	31 (52%)	0.727
Age (years)	71 [61, 80]	69.5 [59.5, 80]	68 [59, 78]	75.5 [63.5, 82]	0.0198
Primary diagnosis (ICD-10)					0.7514
Anterior STEMI	45 (28%)	13 (27%)	13 (26%)	19 (32%)	
Inferior STEMI	37 (23%)	10 (21%)	15 (30%)	12 (20%)	
Other location STEMI	9 (7%)	2 (4%)	4 (8%)	3 (5%)	
NSTEMI	67 (42%)	23 (48%)	18 (36%)	26 (43%)	
Arterial hypertension	78 (49%)	24 (50%)	27 (54%)	27 (45%)	0.6393
History of stroke	24 (15%)	7 (15%)	9 (18%)	8 (13%)	0.7863
Chronic obstructive pulmonary disease	12 (8%)	1 (2%)	4 (8%)	7 (12%)	0.1732
Asthma	3 (2%)	0 (0%)	2 (4%)	1 (2%)	0.3445
Diabetes mellitus					0.0987
Type I	3 (2%)	0 (0%)	3 (6%)	0 (0%)	
Type II	30 (19%)	9 (19%)	7 (14%)	14 (23%)	
Chronic kidney disease	35 (22%)	7 (15%)	12 (24%)	16 (27%)	0.3008
Dyslipidemia	92 (58%)	30 (63%)	32 (64%)	30 (50%)	0.2573
Obesity	36 (23%)	14 (29%)	10 (20%)	12 (20%)	0.4502
Smoker					0.2953
Never	90 (57%)	33 (69%)	24 (48%)	33 (55%)	
Former	36 (23%)	7 (15%)	15 (30%)	14 (23%)	
Current	32 (20%)	8 (17%)	11 (22%)	13 (22%)	
KILLIP class					0.669
I	7 (4%)	1 (2%)	3 (6%)	3 (5%)	
II	65 (41%)	23 (48%)	17 (34%)	25 (42%)	
III	77 (49%)	22 (46%)	28 (56%)	27 (45%)	
IV	9 (6%)	2 (4%)	2 (4%)	5 (8%)	

*STEMI* ST elevation myocardial infarction; *Obesity* body mass index ≥ 30 kg/m^2^; *Dyslipidemia* fasting low-density lipoprotein cholesterol ≥ 100 mg/dl

Analyses were performed in SAS version 9.4 (Cary, NC). *p* values less than 0.05 were considered statistically significant; we performed Tukey’s pairwise comparisons or Dunn’s post hoc tests for normal or skewed continuous variables, respectively, and we preserved family-wise error in post hoc tests using the Holm–Bonferroni adjustment for categorical analyses.

### Ethical disclosure

We conducted this study in compliance with the ethical standards of the Regional Bioethics Committee of Kaunas, Lithuania (the permission number is BE-2-4), and the World Medical Association Declaration of Helsinki on Ethical Principles for Medical Research Involving Human Subjects.

## Results

**Fig. 1:**
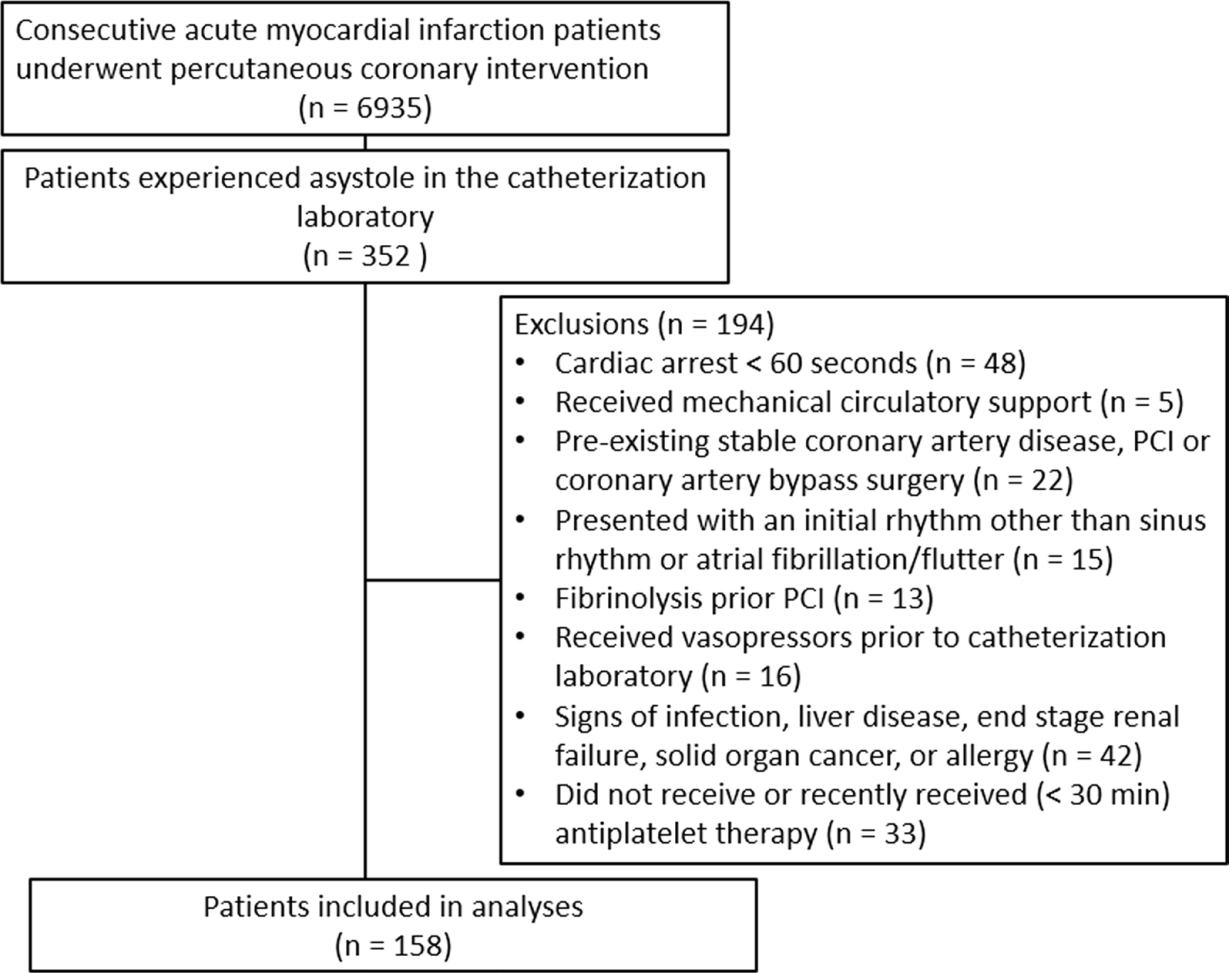
Consort diagram detailing the analysis

There were 158 participants in this study (Fig. 1): 48 (30.4%), 50 (31.6%), and 60 (38.0%) received epinephrine via central IV, IC, and peripheral IV routes, respectively. The median age was 71 [61, 80] years and 56% of the participants were men. Patient characteristics did not differ across administration routes, except for age (higher for peripheral IV than IC route), LVEF (lowest in peripheral IV route), serum potassium (although no significant post hoc differences), hemoglobin (lowest in peripheral IV route), and heart rhythm before cardiopulmonary resuscitation (higher rates of electromechanical dissociation in peripheral IV route) (Tables 1 and 2).

**Table 2:** Heart rhythm, laboratory and instrumental tests of acute myocardial infarction patients undergoing cardiopulmonary resuscitation classified by epinephrine administration route

		Epinephrine administration route			
Instrumental and laboratory test	Overall (*n* = 158)	Central IV (*n* = 48)	Intracoronary (*n* = 50)	Peripheral (*n* = 60)	*p* value
Serum potassium (mEq/L)	4.2 [3.9, 4.6]	4.18 [3.835, 4.39]	4.14 [3.8, 4.6]	4.35 [4.01, 4.63]	0.0415
Hemoglobin (g/l)	134 [120, 147]	136 [128.5, 152.5]	137 [126, 149]	127 [111.5, 141]	0.0066
Platelets (× 10^9^/l)	224.5 [183, 258]	231.5 [185, 273]	216.5 [183, 252]	220 [185.5, 266]	0.8289
International normalized ratio	1.145 [1, 1.3]	1.2 [1.035, 1.3]	1.1 [1, 1.3]	1.15 [1.1, 1.35]	0.3809
Thrombocyte ADP	28 [18, 40]	27.5 [17.5, 36]	25 [17, 40]	29.5 [20, 40]	0.3965
Thrombocyte ADP < 46	137 (87%)	45 (94%)	41 (82%)	51 (85%)	0.204
Left ventricular ejection fraction (%)*	45 [34, 50]	50 [40.5, 51]	45.5 [43, 50]	38.5 [26.5, 48]	0.0006
Heart rhythm before catheterization lab					0.6353
Sinus rhythm	124 (78%)	40 (83%)	38 (76%)	46 (77%)	
Atrial fibrillation	32 (20%)	7 (15%)	11 (22%)	14 (23%)	
Pacemaker	2 (1%)	1 (2%)	1 (2%)	0 (0%)	
Heart rhythm before cardiopulmonary resuscitation					0.0117
Electromechanical dissociation	66 (42%)	15 (31%)	17 (34%)	34 (57%)	
Ventricular fibrillation	92 (58%)	33 (69%)	33 (66%)	26 (43%)	
Electrolyte imbalance prior to catheterization	26 (16%)	8 (17%)	11 (22%)	7 (12%)	0.3464

*ADP* adenosine 5′-diphosphate

*Assessed visually via limited echo prior to cardiac catheterization

**Fig. 2:**
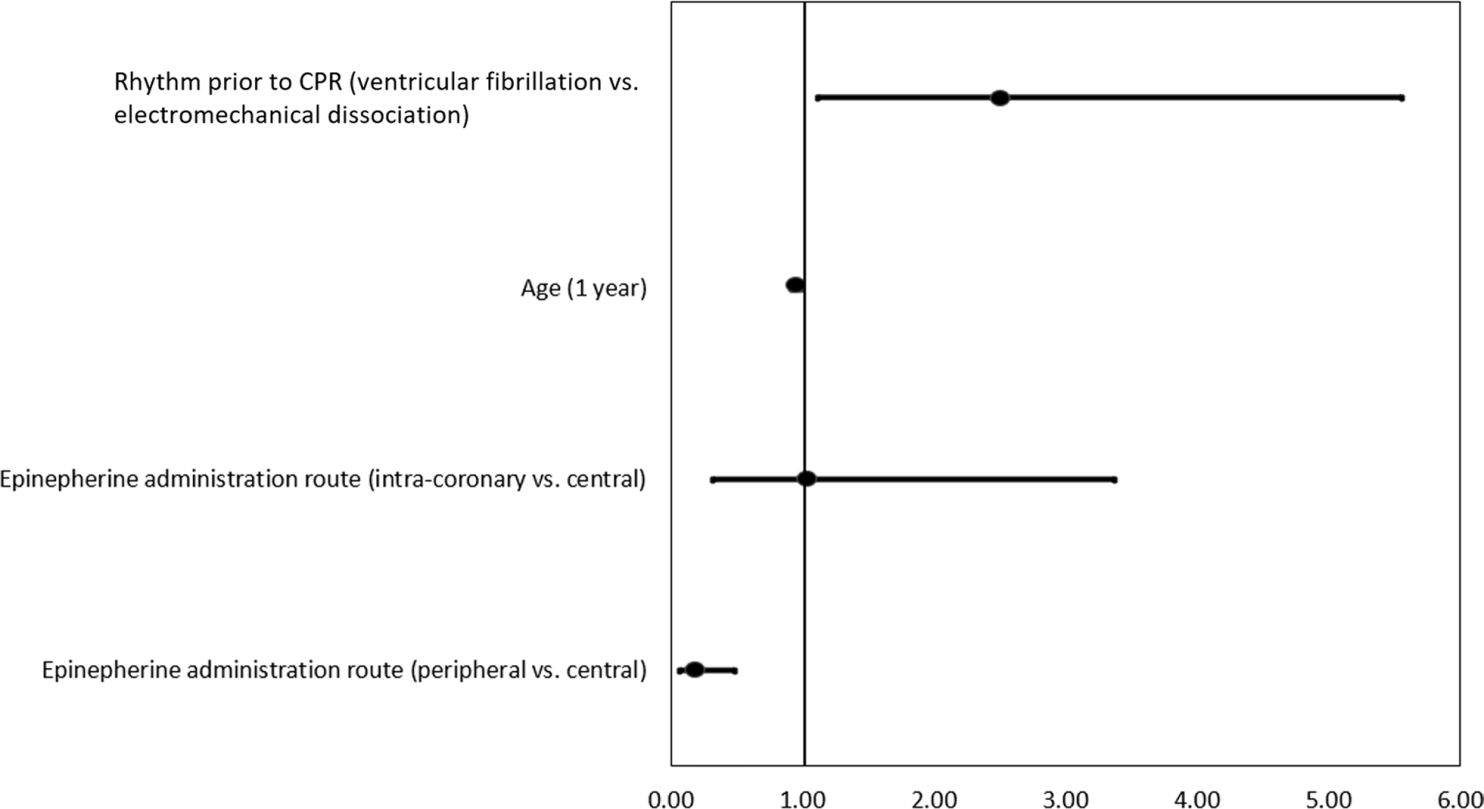
Forest plot for the outcome of return of spontaneous circulation

There were 111 (70%) patients who achieved the primary outcome of ROSC (Table 3). Receiving epinephrine via peripheral IV administration was associated with lower odds of achieving ROSC (OR: 0.14, 95% CI 0.05–0.36, *p* < 0.0001) compared with central IV and IC. (There was no difference between central and IC; *p* = 0.9343.) Epinephrine administration route yielded an area under the receiver operating characteristic curve (AUC) of 0.73, indicating good predictive ability. On multivariable analysis, after adjusting for age and heart rhythm prior to CPR, the peripheral IV route was associated with 5.5-fold lower odds (OR: 0.18, 95% CI 0.07–0.49, *p* = 0.0007) of achieving ROSC compared with the central IV route and there was still no difference between central IV and IC routes (*p* = 0.9516) (Table 4, Fig. 2). Each year increase in age was associated with 5% lower odds of achieving ROSC and that patients with VF instead of EMD prior to CPR had 2.5 times the odds of achieving ROSC (Table 4).

**Table 3:** Clinical course and in-hospital outcomes of acute myocardial infarction patients undergoing cardiopulmonary resuscitation by epinephrine administration route

		Epinephrine administration route			
Clinical course	Overall (*n* = 158)	Central IV (*n* = 48)	Intracoronary (*n* = 50)	Peripheral (*n* = 60)	*p* value
Door-to-catheterization laboratory time (minutes)	26 [20, 34]	26 [20, 35.5]	25.5 [19, 34]	27 [20.5, 33.5]	0.904
Time-to-epinephrine (minutes)	2 [1, 2]	2 [1, 2]	1 [1]	2 [2, 3]	< .0001
Epinephrine dose (mg/ml)	3.5 [2, 8]	3 [2, 5.5]	2 [1, 5]	8 [3, 10]	< .0001
Epinephrine dose (mg/ml)*	3 [1, 4]	3 [1, 4]	2 [1, 4]	3 [2, 6]	0.2143
Number of shocks**	2 [1, 3]	2 [1, 3]	2 [1, 3]	2.5 [2, 4]	0.5424
Return of spontaneous circulation	111 (70%)	41 (85%)	43 (86%)	27 (45%)	< .0001
Time-to-ROSC (minutes)**	10 [7, 16]	10 [7, 15]	10 [5, 16]	15 [8, 27]	0.0855
Stent thrombosis	31 (20%)	5 (10%)	19 (38%)	7 (12%)	0.0004
Stent thrombosis*	31 (27.9%)	5 (12%)	19 (44%)	7 (26%)	0.0004
In-hospital survival	79 (50%)	29 (60%)	38 (76%)	12 (20%)	< .0001
Favorable CPC Score (1–2)***	75 (95%)	29 (100%)	35 (92%)	11 (92%)	0.0674
In-hospital survival with favorable CPC score	75 (47%)	29 (60%)	35 (70%)	11 (18%)	< .0001
Post-arrest left ventricular ejection fraction (%)***	40 [35, 45]	40 [38, 48]	40 [30, 45]	43 [36, 47]	0.1814
Intensive care unit length of stay post-CPR (days)*	2 [1, 3]	2 [1, 2]	2 [2, 3]	1 [1, 2]	0.0004
Intensive care unit length of stay post-CPR (days)***	2 [2, 3]	2 [1, 3]	2 [2, 3]	2 [1, 3]	0.5372

*ROSC* return of spontaneous circulation; *CPC* cerebral performance category; *CPR* cardiopulmonary resuscitation

*For the 111 patients who had ROSC

**For the 92 patients who had VF rhythm prior to CPR

***For the 79 patients who survived

**Table 4:** Adjusted odds ratios for return of spontaneous circulation

Effect	Odds ratio	95% confidence limits		*p* value
Peripheral versus central	0.18	0.07	0.49	0.0007
Intracoronary versus central	1.04	0.32	3.38	0.9516
Age (per 1 year)	0.95	0.91	0.99	0.0083
Rhythm prior to CPR (ventricular fibrillation vs. electromechanical dissociation)	2.49	1.11	5.56	0.0261

*CPR* cardiopulmonary resuscitation

**Fig. 3:**
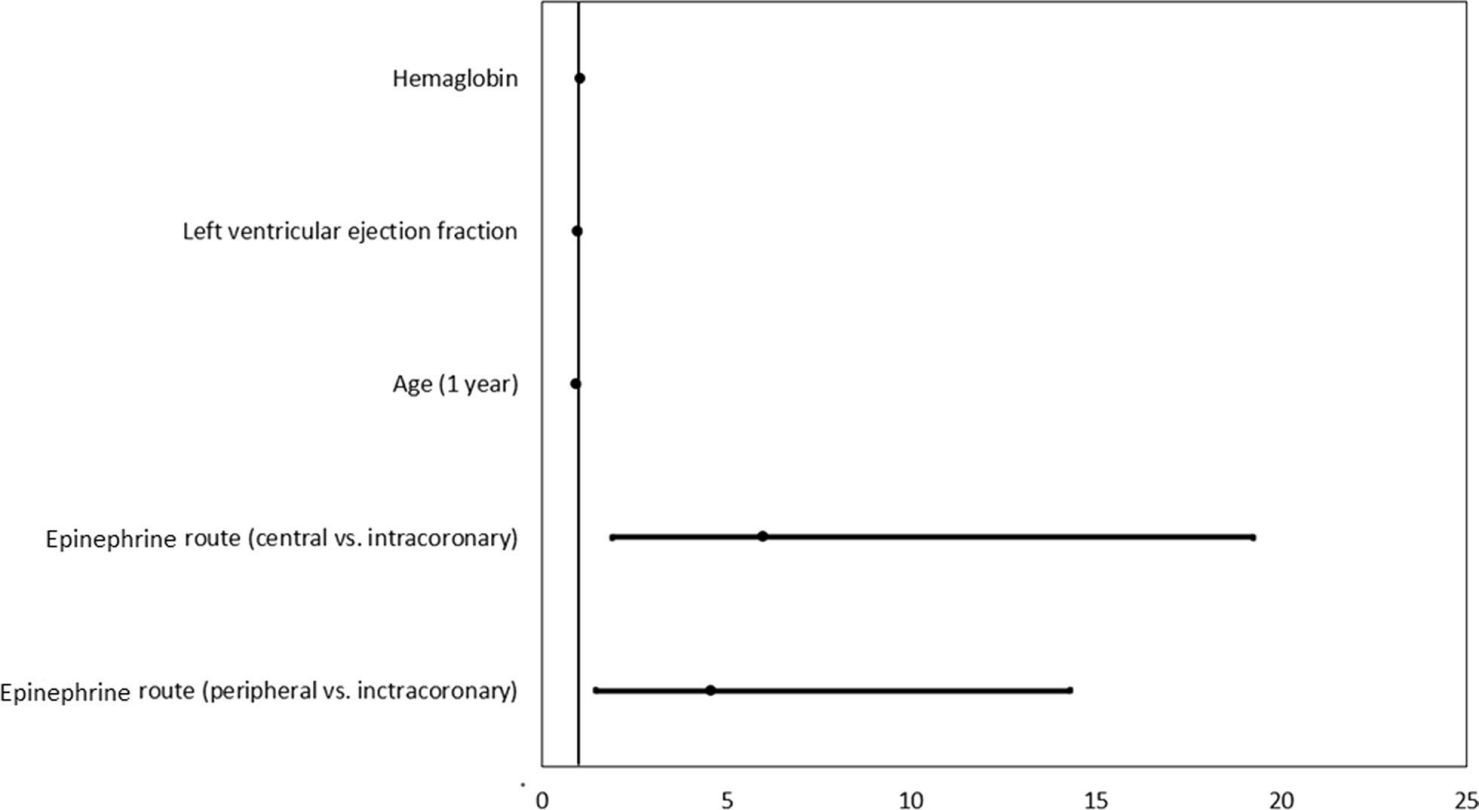
Forest plot for the outcome of stent thrombosis

A total of 31 (20%) patients developed stent thrombosis, 19 of whom had received epinephrine via the IC route (Table 3). This higher risk of thrombosis remained true after adjusting for age, LVEF, and hemoglobin, with the odds of stent thrombosis for patients with IC route being 4.6–6 times higher than for the others (IC vs. peripheral IV OR: 4.6, 95% CI 1.5–14.3, *p* = 0.0094; IC vs. central IV OR: 6.0, 95% CI 1.9–19.2, *p* = 0.0025) (Fig. 3). We performed two sensitivity analyses. The first (not shown) considered thrombosis-specific variables (thrombocyte ADP and platelets) as covariates and confirmed the finding of higher odds of thrombosis with the IC route than in the other two routes. The second considered the outcome of thrombosis, conditional on achieving ROSC. For the 111 patients who achieved ROSC, there was no significant difference in the odds of thrombosis between those who had IC versus peripheral IV administration (OR: 2.3, 95% CI 0.8–6.5, *p* = 0.1277); however, the odds were still nearly sixfold higher compared with central IV administration (OR: 5.7, 95% CI 1.9–17.2, *p* = 0.0022).

A total of 75 (47%) patients survived to discharge and had a good neurologic status; those with peripheral IV administration were the least likely to achieve this outcome (Table 3). After adjusting for age, LVEF, and hemoglobin, the odds of achieving this outcome for those who had IC versus peripheral IV administration were 7.8 (95% CI 2.2–27.0, *p* = 0.0013). There was not a significant difference in the odds for those who had IC versus central (OR = 3.6, 95% CI = 0.96–13.2, *p* = 0.0585).

**Fig. 4:**
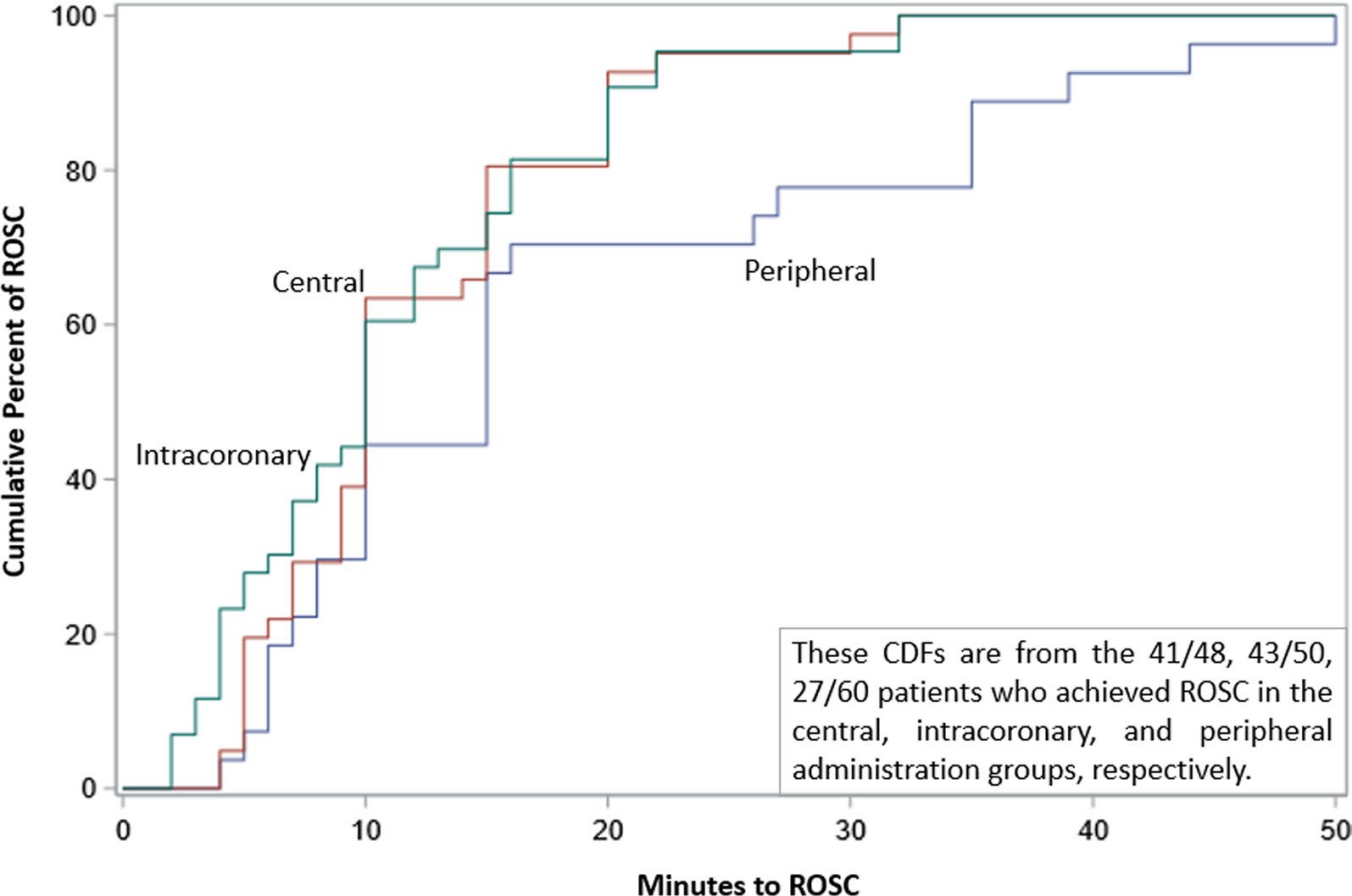
Cumulative density functions (CDFs) comparing time-to-return of spontaneous circulation (ROSC), for those who achieved ROSC

**Fig. 5:**
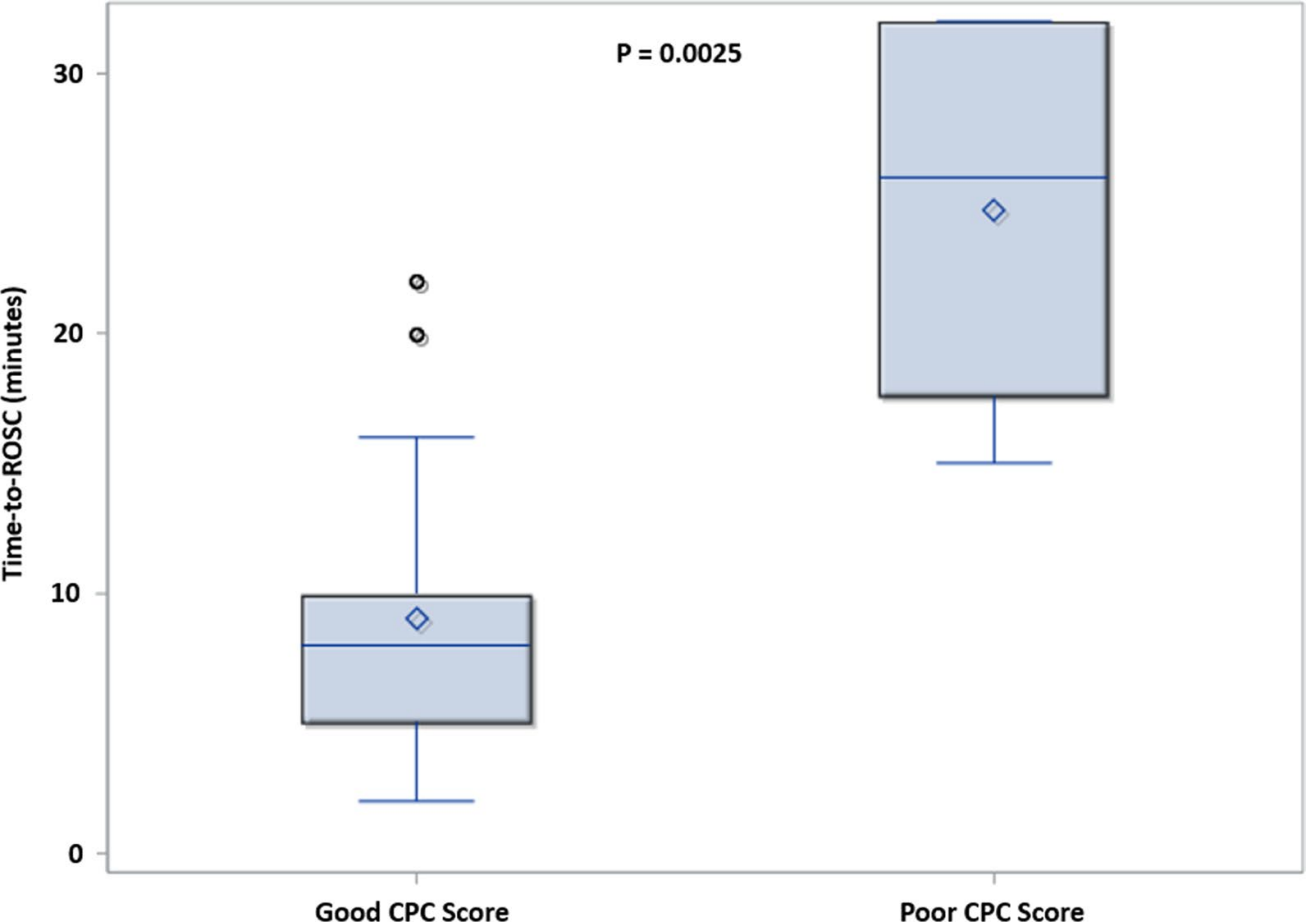
Relationship between time-to-return of spontaneous circulation (ROSC) and CPC score

Finally, we investigated the time-to-ROSC between groups (Fig. 4) and the relationship with favorable neurologic outcomes (CPC score 1–2). Although there were only 4 cases of poor CPC scores, we identified a significant association between time-to-ROSC and CPC score. Specifically, those with good CPC scores had a median time-to-ROSC of 8 [5, 10] minutes compared to 26 [27.5, 32] minutes for those with poor CPC scores (*p* = 0.0025) (Fig. 5).

## Discussion

In this prospective study of 158 patients who presented with NSTEMI or STEMI, suffered cardiac arrest in the catheterization laboratory, and received epinephrine during CPR, we found that the rates of ROSC were substantially higher with central IV and IC epinephrine administration compared to peripheral IV administration. Furthermore, the rates of in-stent thrombosis were higher in the IC route than the other two routes. Central IV and IC epinephrine administration was associated with a higher survival to discharge with a good neurological outcome (CPC score of 1 or 2) compared with the peripheral IV route. Overall, the central IV route was superior as it was equally effective in achieving ROSC as the IC route, while maintaining a lower rate of stent thrombosis.

### The vascular physiology of epinephrine and the role of coronary blood flow

Epinephrine is a catecholamine that binds to *α*1, *β*1, and *β*2 adrenergic receptors in cardiac and smooth muscle tissue. It directs blood flow away from mesenteric circulation and toward skeletal muscle tissue and vital organs via selective arteriolar constriction mediated by *α*1 receptors. Activation of *α*1 receptors results in an overall increase in peripheral resistance which augments aortic pressures and increases venous return. This increases preload and coronary blood flow. Coronary blood flow is further increased by the *β* receptor-mediated relaxation of coronary arteries [24, 25]. While it is unclear whether epinephrine bolsters microcirculation and tissue oxygenation, coronary blood flow appears critical to achieving ROSC [26, 27]. A study of 100 patients with out-of-hospital cardiac arrest measured coronary perfusion pressure via pressure catheters. Patients who achieved ROSC had a significantly higher maximal coronary perfusion pressure than those who did not achieve ROSC (25.6 ± 7.7 vs 8.4 ± 10.1, *p* < 0.001) [28]. Epinephrine is unlikely to bolster coronary blood flow unless it circulates effectively.

### Systemic blood flow and drug circulation during external chest compressions

One reason for the poor performance of the peripheral IV group may be the failure of epinephrine to reach the systemic circulation. During normal flow states, drugs can be given via both peripheral and central routes effectively [29]. During cardiac arrest, however, cardiac output is dramatically reduced with the redistribution of blood flow [30]. Two proposed mechanisms describe the movement of blood flow during external chest compressions. The ‘cardiac pump’ mechanism proposes that direct compression of the ventricles generates antegrade flow, while the ‘thoracic pump’ mechanism proposes that dynamic changes in intrathoracic pressures during chest compression drive blood flow. Prior studies suggest that both proposed mechanisms are responsible for blood flow during CPR [31].

Echocardiographic data suggest that retrograde flow commonly occurs due to the incomplete closure of the atrioventricular valves, regardless of the predominant mechanism of blood flow [32–34]. Tricuspid regurgitation, combined with the local veno-constrictive effect of peripherally administered epinephrine, and an increase in circulation time due to decreased cardiac output during CPR work synergistically to greatly reduce the amount of epinephrine reaching the systemic circulation [30, 35].

Several studies comparing simulated drug delivery via central IV and peripheral IV routes during CPR have found a significant reduction in the time to rise to half of the left ventricular peak concentration with the central IV administration compared to peripheral IV administration [36]. These studies, along with our analysis, suggest that central IV drug administration is a superior method of drug administration during CPR compared with peripheral IV administration [29, 30, 36].

### Epinephrine administration during CPR and neurological outcomes

Data regarding neurological outcomes with epinephrine use during in-hospital CPR are sparse. The Prehospital Assessment of the Role of Adrenaline: Measuring the Effectiveness of Drug administration In Cardiac arrest II trial was a randomized double-blind trial comparing epinephrine to placebo in 8014 patients with out-of-hospital cardiac arrest [37]. It found that epinephrine resulted in a higher rate of ROSC and 30-day survival, but that survivors had worse neurological outcomes than those in the placebo group. However, epinephrine was administered over 20 min after the ambulance was called. Given that neuronal death can occur within minutes, it is difficult to conclusively attribute worse neurological outcomes to epinephrine administration alone.

While the nature of cardiac arrest makes it difficult to conduct high-quality randomized controlled trials on this subject, other studies indicate that epinephrine may have a beneficial effect on outcomes. A large observational study of 119,639 patients with an observed out-of-hospital cardiac arrest found that early epinephrine administration (within 5–18 min of emergency call) was associated with better neurological outcomes than later epinephrine administration [38]. A retrospective study utilizing the Get With The Guidelines-Resuscitation database of 25,095 patients with non-shockable in-hospital cardiac arrest found that the administration of epinephrine had a significant impact on outcomes [39]. When examining the data in 3-min intervals, there was an associated decrease in ROSC and 24-h survival for patients who received epinephrine within 4–6 min, 7–9 min, or > 9 min after cardiac arrest compared to receipt within 1–3 min. Similarly, survival with good neurological function (CPC score 1–2) decreased in a stepwise manner if epinephrine was administered 7–9 min or > 9 min when compared with 1–3 min or 3–6 min. These studies are concordant with our findings.

Conversely, another study from the same registry examined 2978 patients with shockable in-hospital cardiac arrest who underwent defibrillation within the first 2 minutes of CPR [40]. Epinephrine administered within 2 minutes of defibrillation was associated with decreased rates of ROSC, survival to hospital discharge, and survival with good neurological function (CPC score 1–2). This may be due to increased myocardial oxygen consumption or degeneration of a shockable rhythm to PEA arrest from *β* receptor activation by epinephrine [41]. Unfortunately, our study design did not allow us to repeat this analysis.

### Intracoronary epinephrine administration and stent thrombosis

We found that IC epinephrine was associated with substantially higher rates of stent thrombosis, even when adjusting for platelet and thrombocyte ADP levels. The INR did not differ between the three groups, and all patients were on dual antiplatelet medications. Multiple prior case reports describe epinephrine-associated stent thrombosis in anaphylaxis patients [42–45]. Epinephrine increases platelet aggregation, in part by increasing thromboxane *A*_2_ synthesis and synergistically increasing ADP binding to its target receptors [46]. Epinephrine has also been shown to decrease the rate of fibrinolysis, further promulgating a procoagulant environment [47]. Even at low doses, epinephrine counteracts the effect of both aspirin, which decreases thromboxane *A*_2_ levels by inhibiting cyclooxygenase 1 and 2, and P2Y12 receptor inhibitors, as P2Y12 receptors are activated by ADP binding [48]. Most likely, IC epinephrine reached the super-therapeutic level, decreasing the efficacy of antiplatelet medications and increasing the risk of stent thrombosis [47]. While this did not decrease the rate of ROSC, survival-to-discharge, or neurological outcomes, these findings highlight the need for more investigation of the impact of IC epinephrine injection on thrombosis.

### Limitations

The most notable limitation of this study is the lack of treatment randomization. The effort made to limit cofounders by expanding the exclusion criteria resulted in a smaller sample size; of 352 patients experiencing cardiac arrest at the catheterization laboratory, only 152 qualified for inclusion in the analysis. The multicenter design of this study helps to improve the generalizability of findings. Because there were only two centers, we were unable to consider the center as a random effect in the model. However, we observed few differences in patient/treatment characteristics between the two centers and the center was nonsignificant on multivariable analysis. We only studied patients with AMI who had a cardiac arrest in the cardiac catheterization laboratory; hence, our findings may not apply to patients without AMI or patients with OHCA.

## Conclusion

Current guidelines do not specify the route of administration of epinephrine during CPR, and it is typically given through a peripheral IV. Our study suggests significant benefits of delivering epinephrine via the central IV or IC routes, rather than via a peripheral IV route, in terms of the rates of ROSC and survival to hospital discharge. These findings support obtaining a central IV line for patients being administered to the catheterization laboratory with a higher risk of developing cardiac arrest. If central IV access is not obtained prior to cardiac arrest, our findings support the administration of epinephrine via the IC route instead of the peripheral IV route. However, IC administration was associated with a higher risk of stent thrombosis. Future randomized trials comparing these routes are needed to replicate our findings and further investigate the relationship between IC epinephrine and coronary thrombotic events.

## Data Availability

The datasets used in this study are available from the corresponding author upon reasonable request.
